# Functionalized Multi-Walled Carbon Nanotube–Based Aptasensors for Diclofenac Detection

**DOI:** 10.3389/fchem.2021.812909

**Published:** 2022-01-24

**Authors:** Yi Zou, Sophie Griveau, Armelle Ringuedé, Fethi Bedioui, Cyrille Richard, Cyrine Slim

**Affiliations:** ^1^ Institute of Chemistry for Life and Health Sciences (i-CLeHS), Chimie ParisTech, PSL Research University, CNRS, Paris, France; ^2^ Institut de Recherche de Chimie de Paris (IRCP), PSL Research University, CNRS, Chimie ParisTech, Paris, France; ^3^ Université de Paris, CNRS, INSERM, UTCBS, Paris, France

**Keywords:** electrochemical aptasensor, aptamer, functionalized multi-walled carbon nanotubes, diclofenac, impedimetric detection

## Abstract

Driven by the increasing concern about the risk of diclofenac (DCF) residues as water pollutants in the aqueous environment and the growing need for its trace determination, a simple but sensitive electrochemical aptasensor for the trace detection of DCF was developed. To construct the aptasensor, the amine-terminated DCF aptamer was covalently immobilized on the surface of the carboxylic acid–functionalized multi-walled carbon nanotube (f-MWCNT)–modified glassy carbon electrode (GCE) through EDC/NHS chemistry. The f-MWCNTs provide a reliable matrix for aptamer immobilization with high grafting density, while the aptamer serves as a biorecognition probe for DCF. The obtained aptasensor was incubated with DCF solutions at different concentrations and was then investigated by electrochemical impedance spectroscopy (EIS). It displays two linear ranges of concentration for DCF detection, from 250 fM to 1pM and from 1 pM to 500 nM with an extremely low detection limit of 162 fM. Also, the developed biosensor shows great reproducibility, acceptable stability, and reliable selectivity. Therefore, it offers a simple but effective aptasensor construction strategy for trace detection of DCF and is anticipated to show great potential for environmental applications.

## Introduction

Diclofenac (DCF), or 2-(2-(2, 6-dichlorophenylamino) phenyl) acetic acid, is one of the most commonly used non-steroidal anti-inflammatory drugs (NSAID) with great analgesic, antipyretic, and anti-inflammatory properties (Gan, 2010; Altman et al., 2015). DCF has been extensively employed to treat musculoskeletal injuries, arthritis, and post-trauma inflammation (Cannon et al., 2006; Gorissen et al., 2012). Sold under the brand name Voltaren®, among others, DCF is prescribed as oral tablets or a topical gel frequently for rheumatic complaints, acute joint inflammation, and mild to moderate pain. Studies reported that only 6–7% of the topical gel is absorbed, while the rest is washed off the skin or attaches to clothing (Vieno and Sillanpää., 2014). This is significant regarding environmental contamination because a large percentage of the topically applied DCL will end up washed down household drains, ultimately ending up in the wastewater treatment plant (WWTP) influent. The environmental effects of DCL are harmful (Cleuvers, 2004; Haap et al., 2008), and it was included in the watch list of substances in the European Union that requires its environmental monitoring (Richardson and Ternes, 2011). It has been reported that DCF can harm lots of aquatic environment species at a low concentration around 3 nM (Vieno and Sillanpää, 2014). Due to its extensive use, poor degradation, and incomplete elimination during wastewater treatment (Lindqvist et al., 2005; Bonnefille et al., 2018; Grandclément et al., 2020), DCF is often found at various concentration levels in many freshwater ecosystems worldwide (Acuña et al., 2015). Also, its ecotoxicity is due to continuous accumulation in the aquatic environment, and little is known on the long-term effects (Fent et al., 2006; Ort et al., 2009). Considering its negative impact on the organisms and environment, it is crucial and urgent to establish an effective method to quantify DCF in the aquatic environment at trace amounts not only to assess its environmental risk but also to verify and improve the wastewater treatment processes.

Plenty of methods have been reported for DCF quantification in biological fluids and the environment. They are mainly based on conventional methods including high-performance liquid chromatography (HPLC) (Arcelloni et al., 2001), high-performance liquid chromatography–mass spectrometry (HPLC–MS) (Abdel-Hamid et al., 2001), spectrofluorometry (Castillo and Bruzzone, 2006), gas chromatography (Sioufi et al., 1991), thin layer chromatography (Thongchai et al., 2006), and capillary zone electrophoresis (CZE) (Jin and Zhang, 2000). Although these analytic methods are robust, they require a complicated preparation of analytical samples, skillful technicians, the use of organic solvents, and expensive apparatus. In the past few decades, the interests for DCF quantification have been switched to the electrochemical methods owing to their high sensitivity, fast response, low limit of detection (LOD), high dynamic range, user-friendly operation, lower cost, and good portability. Many groups have developed innovative sensors and biosensors for sensitive detection of DCF in various samples using the electrochemical methods (Boumya et al., 2021; Kalambate et al., 2021) such as amperometry (Shalauddin et al., 2019; Slim et al., 2019; Rohani et al., 2021), potentiometry (Lenik, 2014; Elbalkiny et al., 2019), and impedimetric methods (Derikvand et al., 2016; Kassahun et al., 2020).

Carbon nanotubes (CNTs) are nanomaterials with broad applications that are produced on a large scale. It can offer unique properties, such as high surface-to-volume ratio, strong adsorptive ability, high electrical conductivity, and chemical stability (Wang, 2005; Merkoçi, 2006). Specific forms of CNTs, for example, multi-walled carbon nanotubes (MWCNTs) are highly conductive which have been increasingly employed in the construction of electrochemical sensors for signal amplification (Carrara et al., 2014; Power et al., 2018). Besides, it also can be the base matrix for the immobilization of biomolecules to fabricate biosensors. Bioreceptors, such as aptamers and antibodies, can provide the biosensor with high selectivity since they give the particular affinity for the specific targets. Aptamers, mostly artificial single-stranded DNA molecules, are capable of binding targets with a selective and high affinity. In comparison to traditional antibodies, aptamers show advantages such as low cost, thermal stability, easy functionalization, and long shelf time (Song et al., 2008). Recent studies (Jarczewska et al., 2016; Grabowska et al., 2018; Li et al., 2019) showed that there is an increasing interest to develop electrochemical aptasensors for target detection.

In this work, we have integrated the advantages of the MWCNTs to be the matrix providing high grafting density of aptamers, specific biorecognition properties of the aptamers, and electrochemical methods for selective and sensitive quantification of DCF. To fabricate the aptasensor, the glassy carbon electrode (GCE) was first modified with carboxylic acid–functionalized multi-walled carbon nanotubes (f-MWCNTs), followed by the covalent immobilization of amine-terminated DCF aptamer through the formation of amide bonds *via* two-stage EDC/NHS chemistry. The aptamer covalent immobilization was confirmed by ATR−FTIR. Upon the addition of DCF as the target, the aptamer binds the DCF forming the DCF–aptamer complex on the surface of the aptasensor which can directly change the charge transfer resistance (R_ct_) characterized by EIS. In addition, other important performances, such as reproducibility, stability, and selectivity were also evaluated electrochemically.

## Experiment

### Materials and Reagents

Potassium ferrocyanide [K_4_Fe(CN)_6_], 2-[(2,6-Dichlorophenyl)amino] benzeneacetic acid sodium salt (DCF), N-hydroxysuccinimide (NHS), 1-ethyl-3-(3-dimethylaminopropyl) carbodiimide (EDC), and phosphate buffered saline (PBS) were purchased from Sigma-Aldrich and were used as received. The aptamer used for sensing [amine-terminated DCF aptamer (75 bases)]has the sequence of 5′-/5AmMC6/ATA CCA GCT TAT TCA ATT GCA ACG TGG CGG TCA GTC AGC GGG TGG TGG GTT CGG TCC AGA TAG TAA GTG CAA TCT-3′ and was purchased from Integrated DNA Technologies. The DCF aptamer was received as a lyophilized powder, then dissolved in 7.5 ml phosphate buffer solution (0.1 M, pH = 7.4) to make a stock solution of 100 μM DCF aptamer and stored in a freezer at −20 °C until use. Distilled water (18.2 MΩ cm) purified using a Purelab flex system (Elga Water, Veolia, France) was used throughout the experiments for aqueous solution preparation. For the detection of DCF in the PBS (0.1 M, pH = 7.4), a stock solution of 1 mM DCF was weekly prepared and diluted to different concentrations.

### Instruments

All electrochemical measurements including cyclic voltammetry (CV) and electrochemical impedance spectroscopy (EIS) were carried out using a Bio-Logic instrument (Bio-Logic SP-300, driven by EC-Lab software, France). A conventional three-electrode system was used, consisting of a bare or modified glassy carbon electrode (GCE) as the working electrode (3 mm in diameter), an Ag/AgCl electrode as the reference electrode, and a platinum wire as the counter electrode. The surface of the aptasensor was characterized by ATR-FTIR using Agilent Cary 660 to confirm the formation of amide bonds.

### Synthesis of f-MWCNTs

The preparation of MWCNTs/GCE from pristine MWCNT was performed, as described in the study (Karthik et al., 2017), with minor modifications. The pristine MWCNTs (100 mg) were dispersed in 40 mL of a mixture of nitric acid and sulfuric acid (1:3) and heated at 90°C for 4 h. Then, the pristine f-MWCNTs dispersion was diluted to 1 L with mQ water. After this, the dispersion was centrifuged at 4600 rpm for 10 min, and the supernatant was washed until the pH becomes neutral. Suspensions of f-MWCNTs at 5.5 mg/mL are obtained and stored at 4°C in distilled water until use.

### Construction of the Biosensor

**FIGURE 1:**
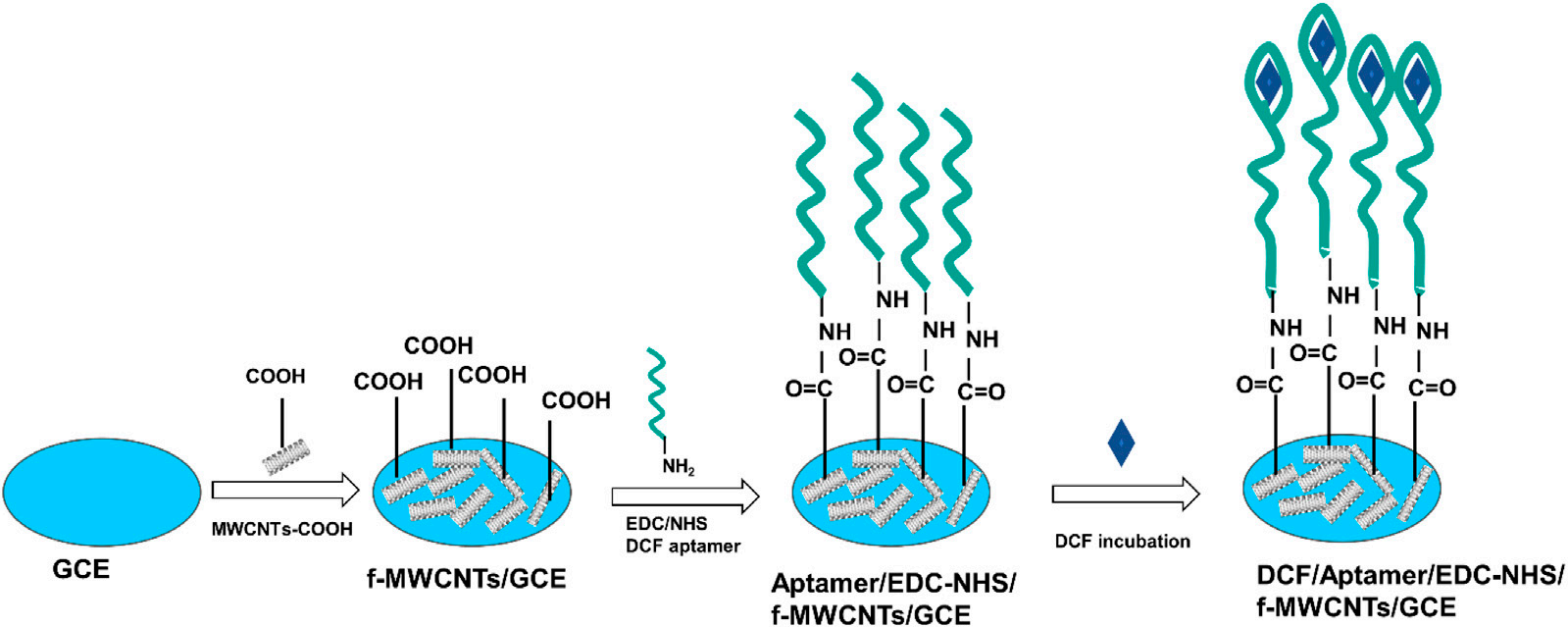
Schematic view of the DCF electrochemical biosensor development.

The process of the aptasensor development is illustrated in Figure 1. Prior to the modification, GCE was polished carefully on grit sandpapers with different grain sizes (1200–2400 and finally 4000) and washed with ultrapure water to remove adherent substances and reached a mirror-like surface. A 5 µL of 1 mg mL^−1^ f-MWCNT suspension was then drop-casted onto the surface of GCE and left to dry in an oven at 70°C for 1 h. The obtained modified electrode is denoted as f-MWCNTs/GCE. After this, the covalent immobilization of the amine-terminated DCF aptamer on the resulting f-MWCNTs/GCE was performed by using a two-stage EDC/NHS chemistry. For this purpose, 10 µL of 50 mM EDC and 100 mM NHS solution in PBS (0.1 M, pH = 6.2) were used to activate the carboxyl groups of f-MWCNTs. After drying in ambient air for 1 h, the obtained activated surface was then incubated with 10 μL DCF aptamer at 10 μM in PBS (0.1 M, pH = 7.4) for 1 h at 25°C (Nazari et al., 2019). Before grafting, the aptamer solution was heated at 75°C for 10 min to denature the single strand allowing for its folding into a complex shape for DCF capture. Once the immobilization of the aptamer was performed, the electrode was washed with PBS to remove the unreacted EDC, NHS, and non-grafted DCF aptamer. Then, the obtained modified electrode was denoted as Aptamer/EDC-NHS/f-MWCNTs/GCE.

To achieve the DCF detection, this aptasensor (Aptamer/EDC-NHS/f-MWCNTs/GCE) was incubated with different DCF concentrations (in 200 μL of PBS 0.1 M, pH 7.4) for 1 h at room temperature and then washed with PBS to eliminate the unreacted DCF.

### Electrochemical Measurements

Electrochemical measurements including cyclic voltammetry (CV) and electrochemical impedance spectroscopy (EIS) techniques were carried out to characterize the modified electrodes at the different steps of the aptasensor construction.

CV was performed in the presence of 5 mM K_4_[Fe(CN)_6_] in PBS (0.1 M, pH = 7.4) by sweeping the potential between −0.4 V and +1.1 V (*vs.* Ag/AgCl) at a scan rate of 50 mV/s.

EIS measurements were performed in K_4_[Fe(CN)_6_] 5 mM in PBS at the open circuit potential (0.16 V) with an oscillation potential of 10 mV over the frequency range of 100 kHz to 10 mHz.

## Results and Discussion

### ATR−FTIR Characterization

**FIGURE 2:**
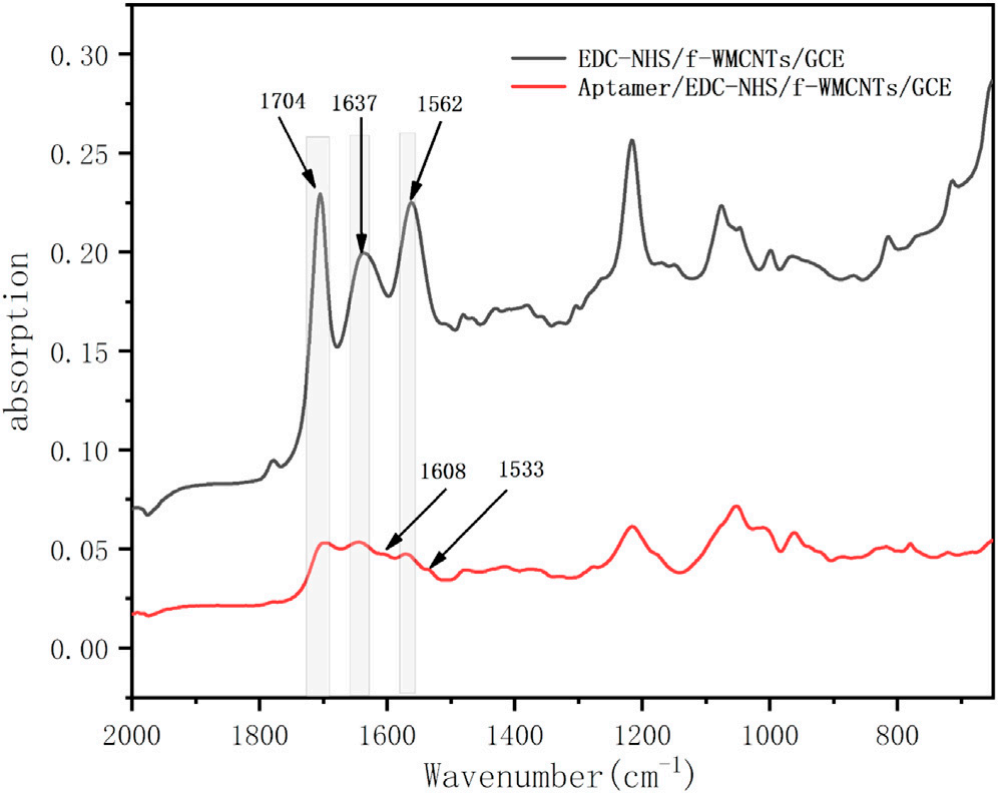
ATR−FTIR spectra of EDC-NHS/f-MWCNTs/GCE (black) and the Aptamer/EDC-NHS/f-MWCNTs/GCE (red) surface.

ATR−FTIR spectroscopy measurements of the EDC-NHS/f-MWCNTs/GCE and Aptamer/EDC-NHS/f-MWCNTs/GCE aptasensor were carried out and presented in Figure 2. For the EDC-NHS/f-MWCNTs/GCE (Figure 2 black curve), the appearance of the peak at 1704 cm^−1^ can be ascribed to the presence of a C=O stretching mode of unreacted carboxylic groups of f-MWCNTs (Pilehvar et al., 2014). Characteristic bands appeared around 1637 cm^−1^ and 1562 cm^−1^ are representative of the COO-NHS ester moiety. After aptamer immobilization, the spectrum of Aptamer/EDC-NHS/f-MWCNTs/GCE (Figure 2 red curve) exhibits a decrease in the peak of the unreacted COOH and COO-NHS ester, while two additional peaks with small intensity appear around 1608 cm^−1^ and 1533 cm^−1^, attributed to the carbonyl stretch (amide II band) and NH bending (amide I band), confirming the successful DCF aptamer immobilization *via* the formation of the amide bond (add references). In addition, two obvious bands around 1220 cm^−1^ and 1080 cm^−1^ are representative of the C-O bond, which are not considered in this work.

### Electrochemical Characterization

**FIGURE 3:**
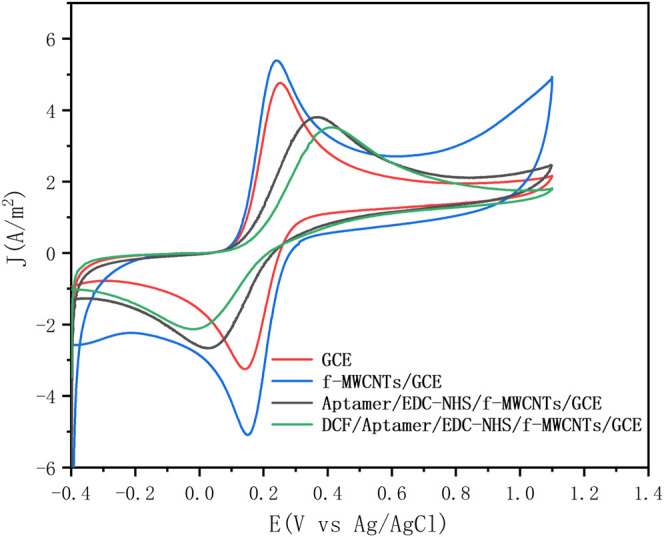
Cyclic voltammograms of the electrode in different stages of the aptasensor construction at 50 mV/s in a solution of PBS (0.1 M, pH = 7.4) containing 5 mM K_4_[Fe(CN)_6_]: bare GCE (red), f-MWCNTs/GCE (blue), Aptamer/EDC-NHS/f-MWCNTs/GCE (black), and DCF/Aptamer/EDC-NHS/f-MWCNTs/GCE (green).

The different steps of the aptasensor construction were also analyzed by cyclic voltammetry (CV). Figure 3 shows the voltammograms of K_4_Fe(CN)_6_ of the carbon electrode at different steps of the aptasensor preparation: bare GCE, f-MWCNT-coated GCE (f-MWCNTs/GCE), Aptamer/EDC-NHS/f-MWCNTs/GCE, and DCF/Aptamer/EDC-NHS/f-MWCNTs/GCE.

It is obvious that modification of GCE with f-MWCNTs leads to an increase in the peak current density of the redox probe (Figure 3 blue curve). This result is due to the special electronic properties of the f-MWCNT material that can significantly accelerate the electron transfer rate *via* its large specific surface area and high conductivity. After the DCF aptamer was covalently immobilized onto f-MWCNT-coated GCE, a significant decrease in the redox peak current density and increased separation potential (Figure 3, black curve, ΔEp = 0.33 V) compared to f-MWCNTs/GCE was observed (Figure 3, blue curve, ΔEp = 0.08 V). This is understandable because non-conductive EDC, NHS, and DCF-aptamer serve as an insulating barrier which impairs the interfacial charge transfer process. After incubating the biosensor with 200 μL of the DCF aptamer at 1 µM concentration for 1 h, the peak current density decreases continually (Figure 3, green curve), indicating that the interaction between the DCF and DCF aptamer forming the DCF/DCF aptamer complex occurs, increasing the resistance of the charge transfer process on the surface of the biosensor.

**FIGURE 4:**
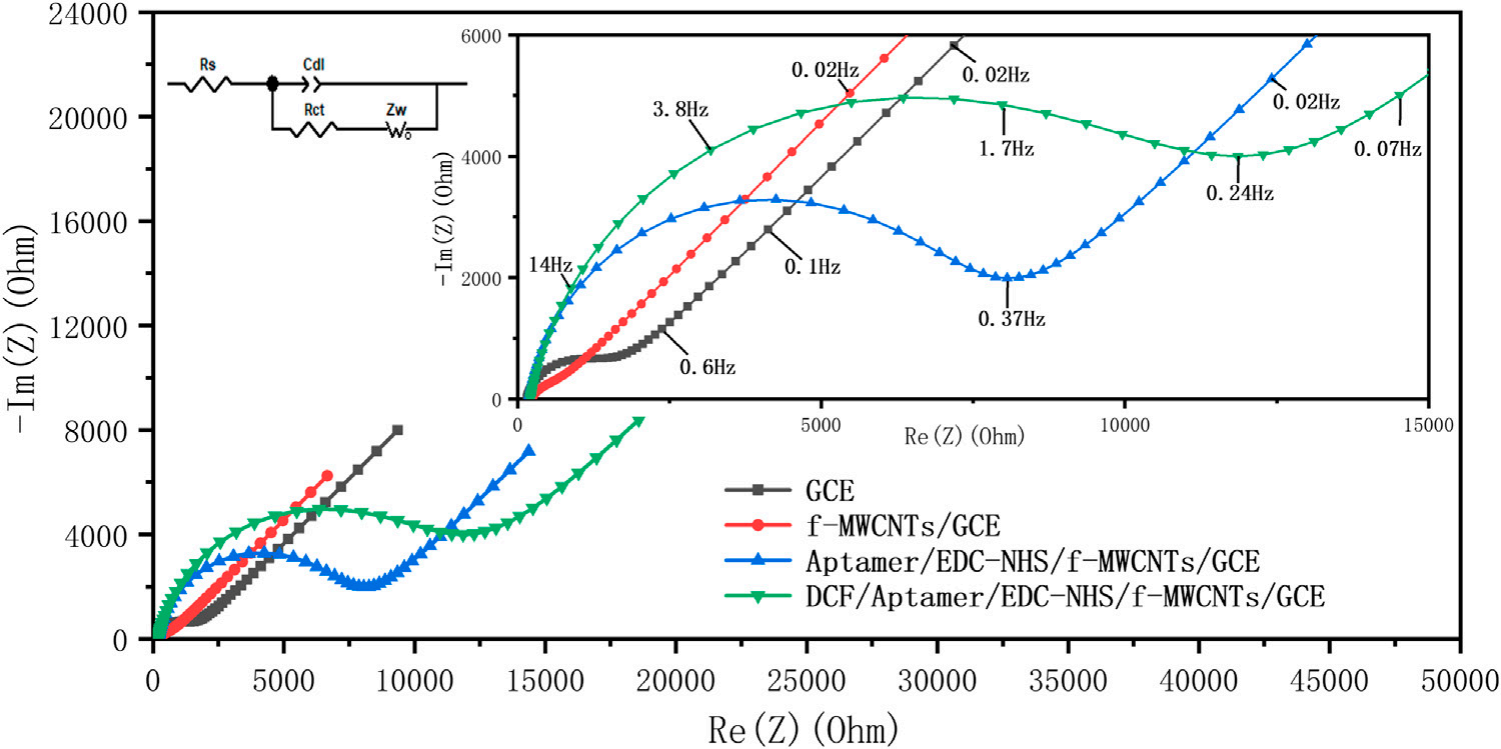
Nyquist plots of: bare GCE (black), f-MWCNTs/GCE (red), Aptamer/EDC-NHS/f-MWCNTs/GCE (blue), and DCF/Aptamer/EDC-NHS/f-MWCNTs/GCE (green) electrodes obtained in 0.1 M PBS (pH = 7.4) containing 5 mM K_4_[Fe(CN)_6_] in the frequency range of 100 kHz to 10 mHz. DCF/Aptamer/EDC-NHS/f-MWCNTs/GCE corresponds to the behavior of the aptasensor after its incubation in a solution containing 1 µM DCF for 1 h at ambient temperature.

Figure 4 shows the Nyquist diagrams obtained in each stepwise fabrication of the aptasensor. The Nyquist plots of the electrodes include a semicircle at the highest frequency range and a linear part at the lowest frequencies. The semicircle part represents the charge transfer resistance (R_ct_) at the electrode surface, while the linear part related to diffusion processes is not affected by the aptasensor fabrication. In this work, a classical Randles equivalent circuit was used for calibration measurements (Figure 4, upper left corner). This equivalent circuit includes R_s_, C_dl_, R_ct_, and Z_w_. The R_s_ element is related to the electrolyte resistance, while the C_dl_ element represents the capacitor derived from the electrode interface double layer. The R_ct_ element is correlated with the charge transfer resistance derived from the electrode surfaces. These parameters are affected by the variations occurring on the electrode surface. The Z_w_ element is a specialized electrochemical element representing the diffusion processes which is not considered in the following analysis.

The Nyquist plot of bare GCE exhibits a small semicircle (Figure 4, black curve), indicating a small charge transfer resistance (R_ct_ = 1220 Ω) and a clean GCE surface. After GCE surface modification with f-MWCNTs (Figure 4, red curve), the R_ct_ value decreases in comparison with that obtained for bare GCE (R_ct_ = 865 Ω), depicting an enhanced conductivity at the electrode–electrolyte interface. When the DCF aptamer is linked on the f-MWCNTs/GCE surface, the charge transfer resistance increases (R_ct_ = 8160 Ω) due to the formation of a non-conductive layer prohibiting the charge transfer between the redox probe and the surface of the aptasensor (Figure 4, blue curve). The R_ct_ value is further enlarged (R_ct_ = 12,390 Ω) (Figure 4, green curve) when the aptasensor is incubated in a solution containing 1 µM DCF, suggesting the formation of the DCF/DCF aptamer complex on the electrode surface that essentially hinders the electron transfer reaction of the probe redox. The results obtained from EIS were in good agreement with the results from CV. It is desirable to note that the aptasensor exhibits a great change in the impedimetric signal after the DCF capture.

### Target Detection and Calibration Curve for DCF

**FIGURE 5:**
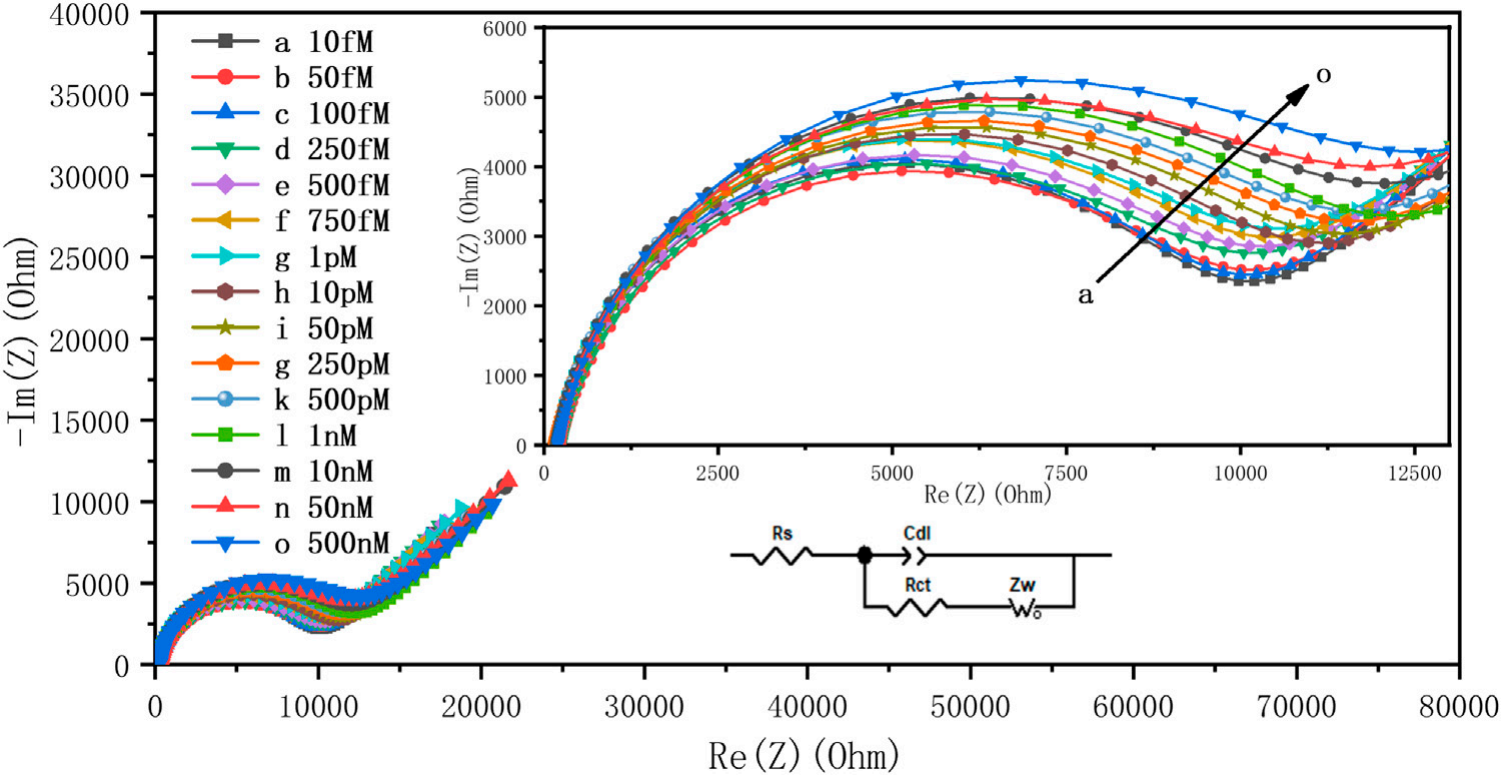
Nyquist plots obtained in 0.1 M PBS containing 5 mM K_4_[Fe(CN)_6_] after incubation of the aptasensor with different concentrations of DCF solutions from 10 fM to 500 nM in the frequency range of 100 kHz to 10 mHz.

The detection of DCF was carried out by incubating the aptamer-modified electrode with DCF solutions at different concentrations, from 5.0 10^−17^ to 10^−14^ M for 1 h. The performances of the aptasensor were investigated by recording the EIS responses in 0.1 M PBS containing 5 mM K_4_[Fe(CN)_6_ ] solution (Figure 5).

The inset of Figure 5 exhibits the Randles equivalent circuit used to fit EIS analysis. As shown, it is clearly noted that the R_ct_ value increases with the increasing concentration of DCF. This can be attributed to the change in the spatial conformation of the aptamer in contact with DCF. With the increase of concentration of DCF, more formation of the DCF/DCF aptamer complex results in a gradual increase of the inhibition of the electron transfer onto the aptasensor surface.

**FIGURE 6:**
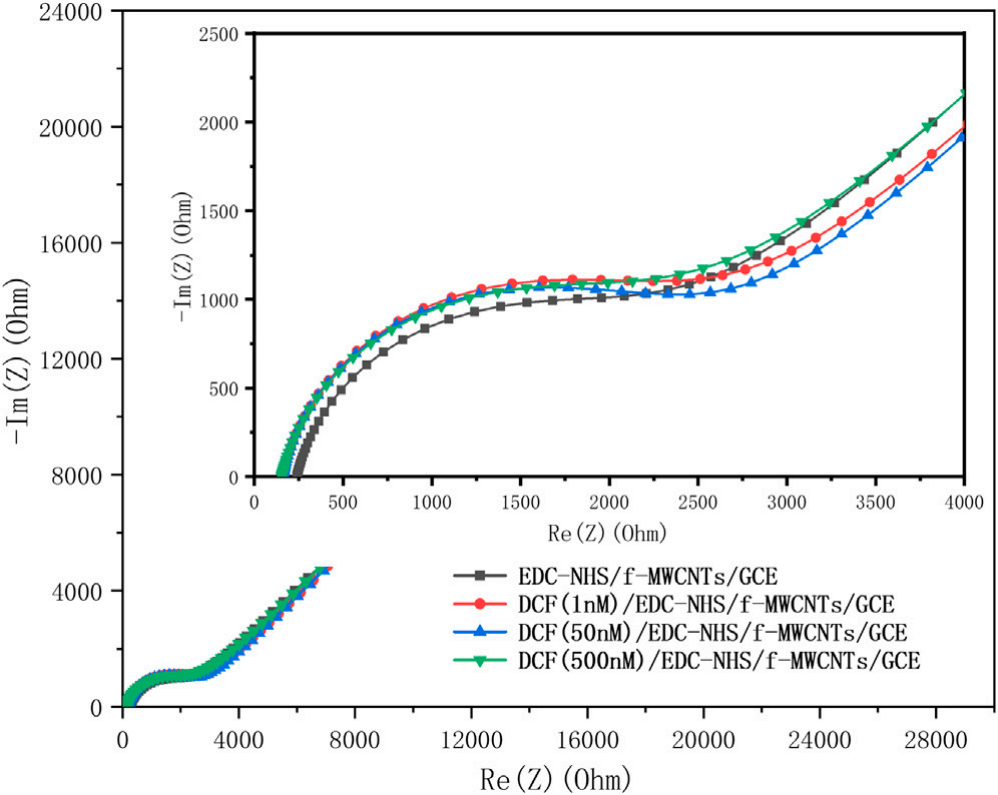
Nyquist plots obtained in 0.1 M PBS containing 5 mM K_4_[Fe(CN)_6_] after incubation of the EDC-NHS/f-MWCNTs/GCE with 1, 50, and 500 nM concentration of DCF in the frequency range of 100 kHz to 10 mHz.

Blank controls were also performed by incubating EDC-NHS/f-MWCNTs/GCE (without aptamer attachment) with different concentrations (1, 50, and 500 nM) of the DCF solution, as illustrated in Figure 6. In the absence of the DCF aptamer, whatever the concentration of DCF, the value of R_ct_ almost keeps constant. This result confirms that the change of R_ct_ for DCF/Aptamer/EDC-NHS/f-MWCNTs/GCE is due to the DCF aptamer instead of other materials or physical adsorption of DCF on the surface of modified GCE. This can also prove indirectly the successful DCF aptamer immobilization on the f-MWCNT/GCE surface.

**FIGURE 7:**
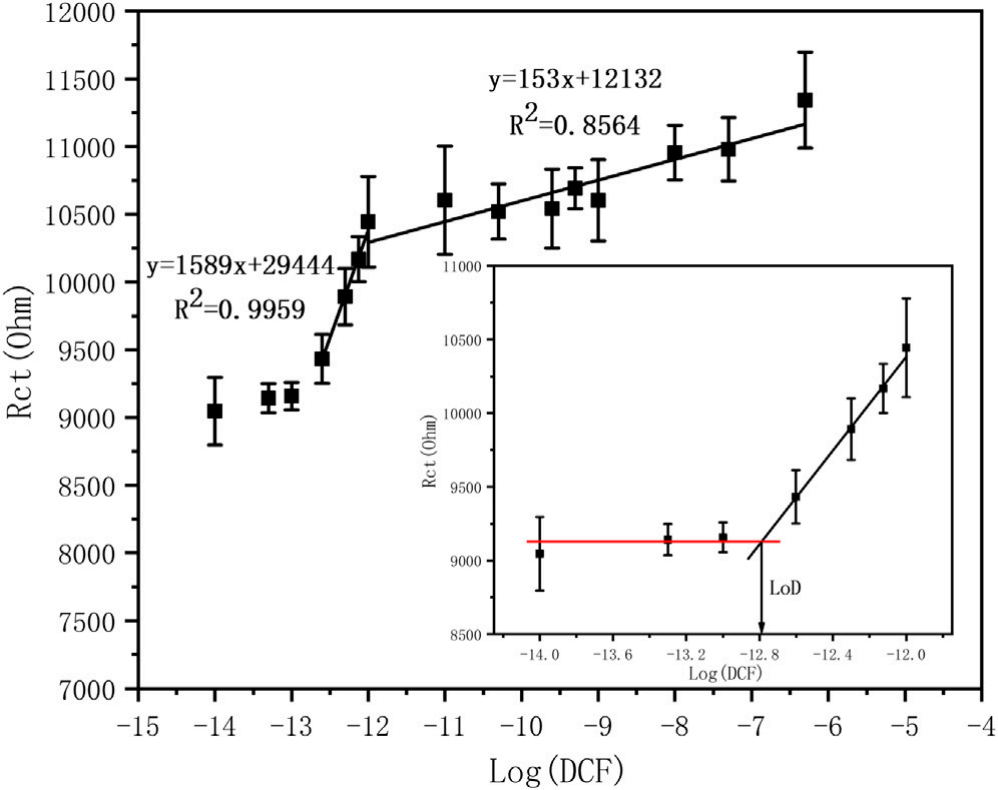
Calibration curve using R_Ct_ *vs.* Log DCF concentration. Inset: LOD determination.

A calibration curve was then deduced from the EIS data of Figure 5. Figure 7 shows the variations of the charge transfer resistance with DCF concentrations. Two regions are observed where the R_ct_ increases linearly with the Log(DCF concentration): from 250 fM to 1 pM and from 1pM to 500 nM.

The regression equation is $R_{ct}=1589\log\;\left[\text{DCF}\right]+29444$ with a correlation coefficient ( $R^{2}$ ) of 0.9959 for low concentrations from 250 fM to 1 pM and $R_{ct}=153\log\;\left[\text{DCF}\right]+12132$ with $R^{2}=0.8564$ for larger concentrations from 1 pM to 500 nM. The detection limit (LOD) was determined from the intersection between the linear part and the non-sensitive linear region (below 250 fM), as shown in the inset of Figure 7. The LOD was calculated to be 162 fM.

### Reproducibility and Stability of the Aptasensor

Once the response of the aptasensor to DCF was demonstrated, the reproducibility and the stability merits of the protocol developed were also evaluated.

**FIGURE 8:**
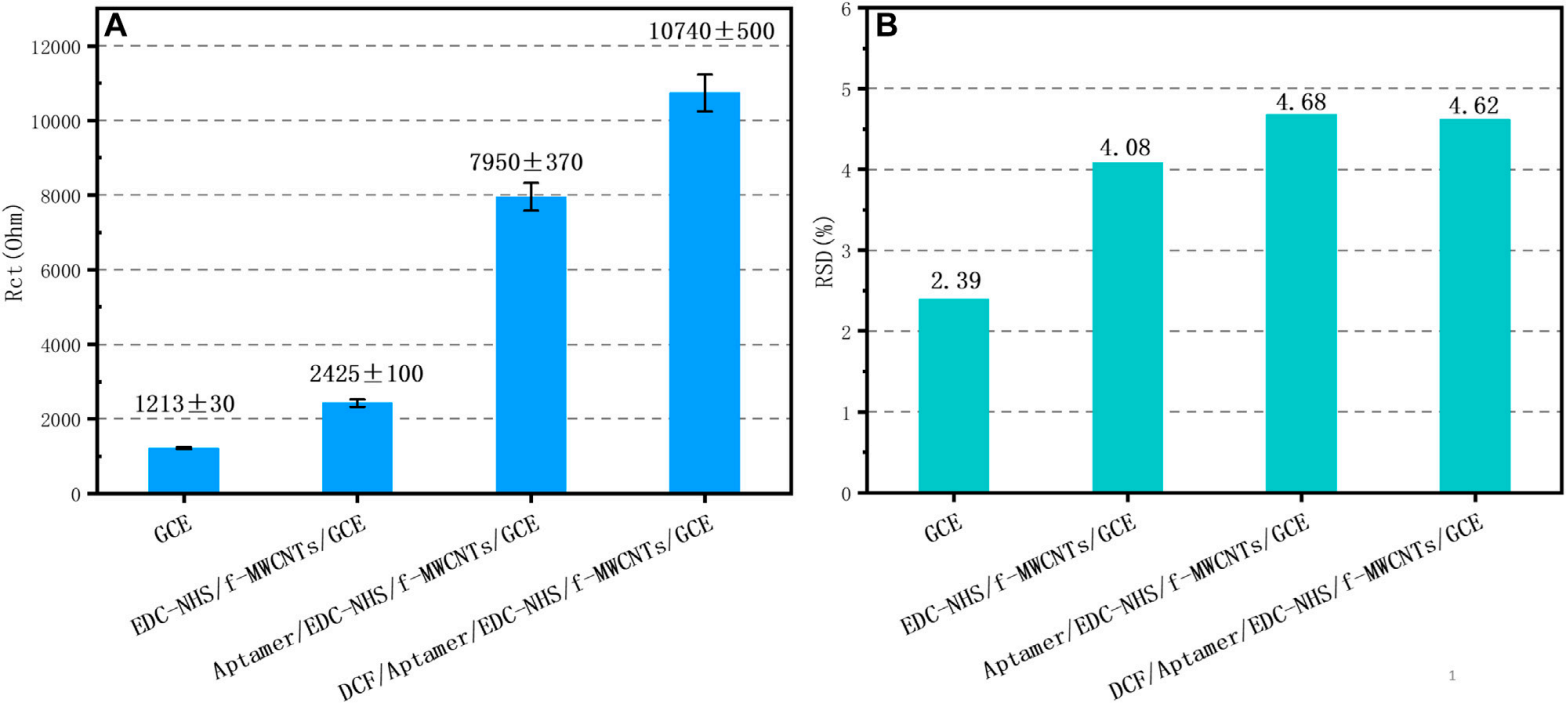
Charge transfer resistance **(A)** and relative standard deviation **(B)** at different steps of the aptasensor construction in PBS (0.1 M, pH = 7.4) containing 5 mM K_4_[Fe(CN)_6_].

Four aptasensors were fabricated; then, R_ct_ values with their relative standard deviations (RSD) were recorded in each step of the aptasensor construction using EIS in 0.1 M PBS containing 5 mM K_4_[Fe(CN)_6_] (Figure 8A). After the fabricated aptasensors were incubated in 500 nM DCF solutions, the RSD of R_ct_ was evaluated to be 4.6%, indicating the reliable reproducibility of the aptasensor (Figure 8B). The variations can be explained by the several uncontrolled factors that can affect the impedimetric results as the electrodes are handmade.

### Interference Studies

**FIGURE 9:**
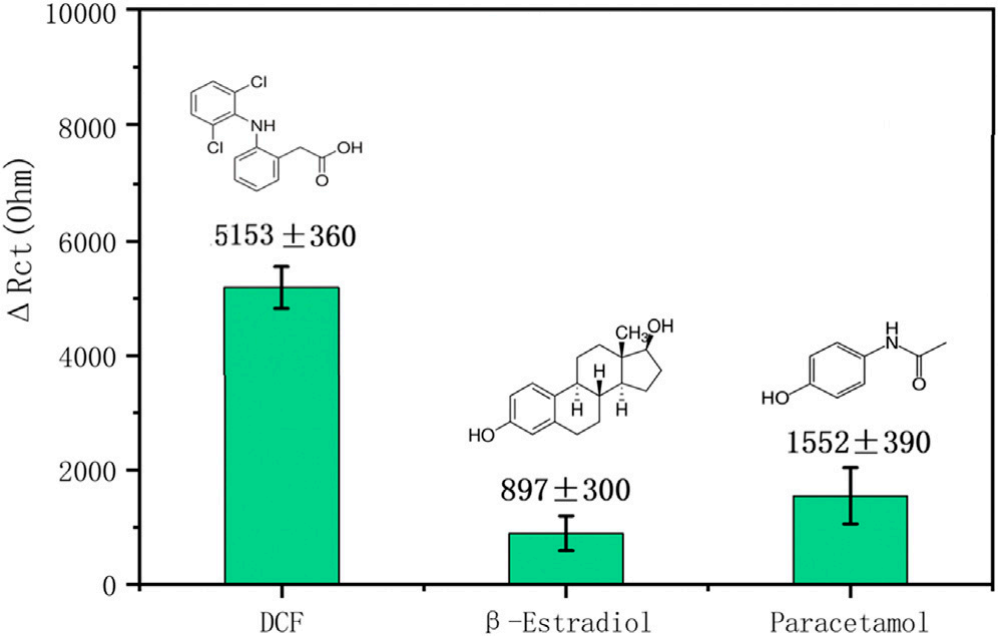
Bar chart of the R_Ct_ changes ( $\Delta R_{Ct}=R_{Ct}\;\left(analyte/apatasensor\right)-R_{Ct}\;\left(aptasensor\right)$ ) of DCF and interferents.

The selectivity of the developed aptasensor was investigated by the EIS signal. The effect of two interferents, paracetamol and *β*-Estradiol, were studied by incubating the developed aptasensors in 1 μM of each interfering substance in 0.1 M PBS at pH 7.4. These two interferents are phenolic compounds with a similar structure as DCF. Figure 9 shows the ΔR_ct_ (difference between the R_ct_ value before and after treatment with the analytes) of DCF and two interferents. The bar chart of the ΔR_ct_ for interfering substances shows small values in comparison with the DCF as the original target. The ΔR_ct_ value for paracetamol is higher than *β*-Estradiol which could be due to its greater physical adsorption on the surface of the biosensor. However, both ΔR_ct_ values for the interferents are lower than that obtained for the target diclofenac. These satisfactory results prove that the newly developed biosensor has good selectivity for distinguishing DCF from other interferents.

### Comparative Data

Up to now, only a few reported electrochemical aptasensors have been developed for DCF detection, as summarized in Table 1. Electrochemical methods include EIS and differential pulse voltammetry (DPV). By adding the DCF aptamer as the biorecognition element, most of these electrochemical biosensors achieved the selective detection of DCF at the nanomolar or picomolar concentration level. The lowest LOD reached with the reported DCF biosensor is 3.4 fM (Azadbakht and Beirnvand, 2017). Compared with the previously reported electrochemical aptasensors for the DCF determination, the aptasensor developed in this work has easier construction steps and an outstanding LOD for DCF determination.

**TABLE 1:** Comparison between performances of the reported electrochemical aptasensors and related techniques for DCF Detection.

Working electrode	Technique	Linearity	LOD (nM)	References
DCF aptamer, gold NPs (AuNPs), and graphene-doped CdS (GR-CdS)modified Au electrode (Aptamer/GR-CdS/Au)	EIS	1–150 nM	0.78	Okoth et al. (2018)
DCF aptamer modified on the surface of the glassy carbon electrode (Aptamer/AHA/GCE)	EIS	0−5μM; 0.001–1 mM	270	Kashefi-Kheyrabadi and Mehrgardi, (2012)
DCF aptamer, acid-oxidized carbon nanotubes (CNT), graphene oxide (GO), and Fe_3_O_4_ nanomaterials modified GCE (Aptamer/CNT/GO/Fe_3_O_4_/GCE)	DPV	100–1300 pM	0.033	Azadbakht and Derikvandi, (2017)
Fe_3_O_4_,AuNP, CNT nanocomposite, amine-terminated DNA sequence (ssDNA1), and DCF-aptamer (ssDNA2) modified GCE (Aptamer/Fe_3_O_4_/AuNP/CNT/GCE)	DPV	0.01−1pM; 10–1300 pM	3.4 × 10^−6^	Azadbakht and Beirnvand, (2017)
DCF aptamer immobilized on the surface of the hydrogel matrix-modified gold electrode (aptamer/hydrogel/SAM/Au)	EIS	30 pM to 1 μM	0.02	Kassahun et al. (2020)
DCF aptamer and f-MWCNTs modified GCE (aptamer/EDC-NHS/f-MWCNTs/GCE)	EIS	0.25–1 pM; 1 pM to 500 nM	1.62 × 10^−4^	This work

## Conclusion

In this work, a novel electrochemical aptasensor for the DCF determination at trace concentration was successfully fabricated. The f-MWCNT nanocomposite and DCF aptamer were attached on the surface of GCE through the drop casting, and covalent amide bonds formed by the carboxylic acid groups on the f-MWCNTs and the amino groups from the DCF aptamer. Through the EIS technique, the addition of DCF shows a change in R_ct_ with the logarithm of DCF concentrations. The densely populated aptamers and the properties to selectively bind the specific target lead to superior properties of the fabricated aptasensor. The obtained aptasensor showed two linear ranges of concentration for DCF detection, from 250 fM to 1pM and from 1 pM to 500 nM with an extremely low detection limit of 162 fM. The developed aptasensor has a reliable reproducibility, stability, and the effect of interfering species on the biosensor is negligible. Its construction is based on a simple design, without complex multi-modification strategies. The modification is achieved with a single nanomaterial easy to deposit and does not require long and complicated steps compared to other biosensors which surface modification combines the use of a mixture of several nanomaterials. Also, the choice of carbon as the electrode material represents several advantages such as the cheapness of the material and the easier cleaning and activation steps required for gold electrodes. The proposed strategy could also be transferred to miniaturized devices or integrated into microfluidic systems for example.

## Data Availability

The raw data supporting the conclusion of this article will be made available by the authors, without undue reservation.
